# Molecular Evolution of Zika Virus during Its Emergence in the 20^th^ Century

**DOI:** 10.1371/journal.pntd.0002636

**Published:** 2014-01-09

**Authors:** Oumar Faye, Caio C. M. Freire, Atila Iamarino, Ousmane Faye, Juliana Velasco C. de Oliveira, Mawlouth Diallo, Paolo M. A. Zanotto, Amadou Alpha Sall

**Affiliations:** 1 Institut Pasteur de Dakar, Dakar, Senegal; 2 Laboratory of Molecular Evolution and Bioinformatics, Department of Microbiology, Biomedical Sciences Institute, University of Sao Paulo, Sao Paulo, Brazil; Centers for Disease Control and Prevention, United States of America

## Abstract

Zika virus (ZIKV) is a mosquito-borne flavivirus first isolated in Uganda in 1947. Although entomological and virologic surveillance have reported ZIKV enzootic activity in diverse countries of Africa and Asia, few human cases were reported until 2007, when a Zika fever epidemic took place in Micronesia. In the context of West Africa, the WHO Collaborating Centre for Arboviruses and Hemorrhagic Fever at Institut Pasteur of Dakar (http://www.pasteur.fr/recherche/banques/CRORA/) reports the periodic circulation of ZIKV since 1968. Despite several reports on ZIKV, the genetic relationships among viral strains from West Africa remain poorly understood. To evaluate the viral spread and its molecular epidemiology, we investigated 37 ZIKV isolates collected from 1968 to 2002 in six localities in Senegal and Côte d'Ivoire. In addition, we included strains from six other countries. Our results suggested that these two countries in West Africa experienced at least two independent introductions of ZIKV during the 20^th^ century, and that apparently these viral lineages were not restricted by mosquito vector species. Moreover, we present evidence that ZIKV has possibly undergone recombination in nature and that a loss of the N154 glycosylation site in the envelope protein was a possible adaptive response to the *Aedes dalzieli* vector.

## Author Summary

Zika fever is a mosquito-borne illness caused by a flavivirus. Human infections with Zika virus (ZIKV) could cause fever, malaise and cutaneous rash. Despite several ZIKV reports since 1947 when it was first isolated at Zika forest in Uganda, molecular evolution of ZIKV as an emerging agent remains poorly understood. Moreover, despite several ZIKV reports from Africa and Asia, few human cases were notified until 2007 when an epidemic took place in Micronesia. In West Africa, surveillance programs have reported periodic circulation of the virus since 1968. To help fill the gap in understanding ZIKV evolution, 43 ZIKV samples were analyzed. We focused on: (*i*) adaptive genetic changes including protein glycosylation patterns, (*ii*) phylogenetic relationship among isolates and their spatiotemporal patterns of spread across Africa and Asia and, (*iii*) dispersion among vertebrate reservoirs and invertebrate vector species. Our results indicated that ZIKV may have experienced recombination in nature and that, after it emerged from Uganda in the early of the 20^th^ century, it moved to West Africa and Asia in the first half of the century, without any clear preference for host and vector species.

## Introduction

Zika virus (ZIKV) is a mosquito-borne flavivirus, a member of the Spondweni serocomplex, whose natural transmission cycle involves mainly vectors from the *Aedes* genus (*A. furcifer*, *A. taylori*, *A. luteocephalus* and *A. africanus*) and monkeys [1], while humans are occasional hosts. Clinical pictures range from asymptomatic cases to an influenza-like syndrome associated to fever, headache, malaise and cutaneous rash [2], [3]. Likewise, direct contact is also considered a potential route of transmission among humans, probably during sexual intercourse [4]. The first isolation of ZIKV was in 1947 from the blood of a sentinel Rhesus monkey No. 766, stationed in the Zika forest, near the Lake Victoria in Uganda, and in 1948 ZIKV was also isolated in the same forest from a pool of *A. africanus* mosquitoes [5]. Thereafter, serological and entomological data indicated ZIKV infections in the African continent in Nigeria in 1971 and 1975 [6], Sierra Leone in 1972 [7], Gabon in 1975 [8], Uganda in 1969 and 1970 [9], Central African Republic in 1979 [10], Senegal from 1988 to 1991 [11] and Côte d'Ivoire in 1999 [12]. Recently, ZIKV was detected in Senegal in 2011 and 2012 (unpublished data). In addition, ZIKV infections in Asia were reported in Pakistan [13], Malaysia [14], Indonesia in 1977 and 1978 [15], Micronesia in 2007 [16], [17] and Cambodia in 2010 [18]. Although ZIKV was repeatedly isolated, only 14 human cases were reported before April 2007, when a Zika fever (ZF) epidemic occurred in Yap island in Micronesia, where 49 confirmed cases and 73% of the residents older than 3 years provided serologic evidence for recent ZIKV infection [16]. This outbreak showcased the potential of ZF as an emerging disease, which could be misdiagnosed as dengue fever, as happened during the beginning of the Micronesian outbreak [16], [17].

The ZIKV genome consists of a single-stranded positive sense RNA molecule with 10794 kb of length with 2 flanking non-coding regions (5′ and 3′ NCR) and a single long open reading frame encoding a polyprotein: 5′-C-prM-E-NS1-NS2A-NS2B-NS3-NS4A-NS4B-NS5-3′, that is cleaved into capsid (C), precursor of membrane (prM), envelope (E) and seven non-structural proteins (NS) [19], [20]. The E protein (≈53 kDa) is the major virion surface protein. E is involved in various aspects of the viral cycle, mediating binding and membrane fusion [21]. The NS5 protein (≈103 kDa) is the largest viral protein whose C-terminal portion has RNA-dependent RNA polymerase (RdRP) activity and the N-terminus is involved in RNA capping by virtue of its processing due to methyl transferase activity [21]. The 3′NCR of the ZIKV genome contains about 428 nucleotides, including 27 folding patterns [20] that may be involved in the recognition by cellular or viral factors, translation, genome stabilization, RNA packaging, or cyclization [21]. Although diverse studies have contributed greatly to our understanding of the evolutionary biology of flaviviruses in general [22]–[25], few studies have addressed ZIKV evolutionary biology [17], [26]. Those studies report three main ZIKV lineages, one from Asia and two from Africa. Aiming to fill this gap and gain better insights ZIKV molecular evolution in the 20^th^ century, we investigated 43 ZIKV strains, sampled from 1947 to 2007 in Africa and Asia, to describe phylogenetic relationships, selective influences, recombination events, phylodynamics, phylogeography, host correlations with viral lineages and glycosylation patterns.

## Methods

### Ethical statements

Samples used in this study are part of the Institute Pasteur in Dakar collection (WHO Collaborating Centre for Arboviruses and/or Hemorrhagic Fever Reference and Research). Monkey and human strains (AnD 30332 and HD 78788) were obtained respectively in 1979 and 1991 in Senegal during routine surveillance. None of the data was directly derived from human or animal samples but rather from cell culture supernatant. Therefore all the samples were anonymous and only reference numbers were used during the analysis that originated this study.

### Virus isolates

ZIKV strains were provided by CRORA at the Institute Pasteur of Dakar. The strains were obtained from mosquitoes, humans and other mammals isolated in Burkina Faso, Central African Republic, Côte d'Ivoire and Senegal in West Africa (Table S1). Viral stocks were prepared by inoculating viral strains into *Aedes pseudoscutellaris* clone 61 monolayer in Leibovitz 15 (L-15) growth medium (GibcoBRL, Grand Island, NY, USA) supplemented with 5% fetal bovine serum (FBS) (GibcoBRL, Grand Island, NY, USA), 10% Tryptose Phosphate and antibiotics (Sigma, Gmbh, Germany). Viral infection was confirmed after seven days of propagation by an indirect immunofluorescence assay (IFA) using specific hyper-immune mouse ascitic fluid, as described previously [27]. Cultures supernatants were collected for virus RNA isolation.

### RNA extraction

RNA was extracted from ZIKV stocks using the QIAamp RNA Viral Kit (Qiagen, Hilden, Germany) according to the manufacturer's recommendations. RNA was eluted in 50 µl of AVE buffer and stored at −80°C until use.

### RT-PCR amplification

For cDNA synthesis, 10 µl of viral RNA was mixed with 1 µl of each of a reverse primer (2 pmol), 1 µl of deoxynucleotide triphospahte (dNTP) (10 mM each dNTP and the mixture was heated at 65°C for 5 min. Reverse transcription was performed in 20 µl mixture containing mixed of 2.5 U RNasin (Promega, Madison, USA) 5 U of Superscript III reverse transcriptase (Invitrogen, Carlsbad, USA) and incubated at 55°C for 50 min, followed by 70°C for 15 min. PCR products were generated independently using the primers Unifor/Unirev, FD3/FU1 and VD8/EMFI to amplify partial E, NS5 and NS5/3′NC region respectively [28]. Five microliters of cDNA were mixed with 10× buffer, 5 µl of each primer, 5 µl of dNTPs 10 mM, 3 µl of MgCl_2_, and 0.5 µl of Taq polymerase (Promega, Madison, USA).

### Nucleotide sequencing

PCR products of the expected size were purified from agarose gels with the QiaQuick Gel Extraction Kit (Qiagen, Hilden, Germany) as specified by the manufacturer. Both strands of each PCR product were sequenced directly with the ABI Prism BigDye Terminator Cycle Sequencing Ready Reaction Kit V3.1 on an Applied Biosystems 3100 DNA Analyzer (Applied Bisoystem, Foster City, CA, USA) at the Laboratory of Molecular Evolution and Bioinformatics, Biomedical Sciences Institute, University of Sao Paulo, Brazil. We deposited thirty two 753 bp-long sequences from the E gene (Accession numbers: KF383015-KF383046), thirty one of NS5 (708 bp) (Accession numbers: KF38304-KF383114), thirty seven of 3′NCR (537 bp) (Accession numbers: KF383047-KF383083) and six genomes (10274 bp) (Accession numbers: KF383115–KF383120) in GenBank (www.ncbi.nlm.nih.gov/genbank/) from thirty eight viral strains (Table S1). Additional sequences representing strains from Kedougou in Senegal, Nigeria, Malaysia, the Ugandan prototype MR766, the strain related to Micronesian outbreak in 2007 and the Spondweni virus were obtained from GenBank, with the following accession numbers, respectively: HQ234501, HQ234500, HQ234499, NC_012532, EU545988 and DQ859064.1 (Table S1).

### Recombination detection

Prior to the analyses, all sequences were aligned with MUSCLE v3.7 [29] and manually edited with SeaView v4.3.3 [30]. To prevent potential biases during phylogenetic inference due to recombination, we first analyzed the sequences of available ZIKV genomes with RDP v4.4.8 program [31] that incorporates RDP [32], GENECONV [33], Chimaera [34], MaxChi [35], Bootscan [36], SiScan [37] and 3Seq [38] methods to uncover evidence for recombination events. Only events with *p*-values≤0.01 that were confirmed by four or more methods were considered, using the Bonferroni correction to prevent false positive results [39], as implemented in the RDP program [31]. In addition, the occurrence of recombination in genomes was also investigated with the Rec-HMM program that estimates breakpoints based on the Phylo-HMM approach, which models tree topology changes over the columns of a multiple alignment [40]. Moreover, potential intra-gene recombination was also inspected with RDP using individual gene datasets, and the incompatibility among phylogenies inferred from genes (NS5 and E) was also investigated with GiRaF v1.01 [41] that searches incompatible clades among posterior set of trees (PST) obtained from different genomic regions with threshold of 70% for incompatible clades. The PST was obtained during Monte Carlo Markov chain (MCMC) stationarity using four chains, one ‘cold’ and three ‘heated’, after 20 million of generations, sampling every 5000 generations using MrBayes v3.2.1 [42].

### Phylogenetic analyses

The phylogenetic signal content of the sequence datasets to phylogenetic reconstruction was investigated by Likelihood mapping method [43], implemented in TREE-PUZZLE v5.2 [44]. The concordance among gene (E and NS5) datasets without recombinant sequences was further assessed using a permutation test with significance level (α) of 0.05 after 10000 permutations, implemented in the Congruence among Distance Matrices (CADM) package [45]. Phylogenetic trees were generated by Maximum Likelihood (ML) criterion using GARLI v2.0 [46] that uses a stochastic algorithm to estimate simultaneously the best topology, branch lengths and substitution model parameters that maximize the log Likelihood (lnL). The confidence of ML trees was assessed by the convergence of lnL scores from ten independent replicates. We used a substitution model based on general time reversible (GTR) model with gamma-distributed rate variation (Γ) and a proportion of invariant sites (I). Support for the topology was obtained after 1000 non-parametric bootstrap replicates with GARLI. Then, we summarized the bootstrap trees into one consensus tree to access bootstrap values, using Dendropy v3.10.1 [47].

### Selection analyses

To infer the selection pressures acting on each gene of ZIKV, we estimated the difference between the non-synonymous (*dN*) and synonymous (*dS*) rates per codon site using the single likelihood ancestor counting (SLAC) algorithm available in HyPhy v0.99 [48], assuming a significance level of 1% (α = 0.01). In the HyPhy output, values of ω are expressed as ω = *dN* - *dS*. Therefore, ω smaller than zero (ω<0) indicate purifying, negative selection.

### Prediction of glycosylation sites

Potential glycosylation sites that may have adaptive value were previously described in ZIKV proteins [17], [20], [26]. Thus, we investigated partial E sequences to detect potential glycosylation sites using NetCGlyc v1.0 [49], NetOGlyc v3.1 [50], YinOYang v1.2 [51] and NetNGlyc v1.0 [51], [52] methods that employ algorithms based in neural networks to predict, respectively, C-mannosylated, mucin-type O-linked, N-acetylglucosamine (GlcNAc) and N-linked glycosylation sites. To infer the structural position of the predicted glycosylation sites, we modeled the tridimensional structures of E regions of viral polyprotein of the Micronesian strain (GenBank accession number ACD75819). We used the homologous sequences from Japanese Encephalitis virus (PDB code 3p54), West Nile virus (PDB code 2i69) and Dengue virus type 3 (PDB code 1uzg). The amino acids sequences were aligned using MUSCLE v3.7 [29], a total of 1000 independent models were generated and optimized using Modeller v9.10 [53], and the best models were validated with PROCHECK v3.5.4 [54].

### Phylodynamic analysis

Maximum Clade Credibility (MCC) trees were inferred using a Markov Chain Monte Carlo (MCMC) Bayesian approach implemented on the program BEAST v1.6.2 [55] under GTR + Γ + I and a relaxed (uncorrelated lognormal) molecular clock [56]. MCMC convergence was obtained for four independent runs with 50 million generations, which were sufficient to obtain a proper sample for the posterior at MCMC stationarity, assessed by effective sample sizes (ESS) above 200 inspected using Tracer v1.5 (http://tree.bio.ed.ac.uk/software/tracer/). Furthermore, using the concatenated sequences of E and NS5 genes, we employed a discrete model attributing state characters representing isolation locality, animal source, recombination and N- linked glycosylation on E protein of each of the strains with the Bayesian Stochastic Search Variable (BSSVS) algorithm [57], implemented in BEAST. This method estimates the most probable state at each node in the MCC trees, allowing us to reconstruct plausible ancestral states on these nodes. Moreover, we represented the viral migration in Google Earth (http://www.google.com/earth/), using the SPREAD v1.0.3 program [58]. We evaluated the correlation among viral states and inferred phylogenies from PST by the parsimony score (PS), association index (AI) and monophyletic clade size (MC), with BaTS v1.0 [59] after 10000 null replications. In addition, we investigated the occurrence of correlated evolutionary change among ZIKV phenotypes (glycosylation pattern and vector host) along PST, employing a ML approach to test the fit of the two evolutionary models, one where the two traits evolve independently on the phylogenetic tree (independent model), and one where they evolve in a correlated way (dependent model) [60], using BayesTraits program (http://www.evolution.rdg.ac.uk/). To evaluate model suitability to ZIKV data, we estimated the marginal likelihoods for both models after 1000 bootstrap replications and compared Bayes factors (BF) between models [61], using Tracer v1.5.

## Results/Discussion

### Recombination among ZIKV strains

The primary analyses with RDP suggested 13 recombination events in ZIKV complete genomes (Table S2), Rec-HMM also detected genomic breakpoints with confidence in the following alignment positions: 1044 to 1095, 5181 to 5238, 9007 to 9132 and 9580 to 9631 (Figure S1). Since the results obtained by both methods revealed breakpoints in the E and NS5 genomic regions, we investigated these evidences with RDP on partial gene sequences. We found a single event in E sequences with estimated breakpoints near to the 414^th^ and 1065^th^ site of E gene reaching nine viral strains: ArA986, HD78788, ArA27101, ArA27290, ArA27096, ArA27443, ArA27407, ArA27106 and ArA982. These results were found by Bootscan, Maxchi, Chimaera, SiSscan and 3Seq methods and supported by significant *p*-values of 1.31E-5, 2.85E-3, 1.59E-3, 1.79E-29 and 6.85E-19, respectively. Likewise, only one recombination event was detected in NS5 sequences with estimated breakpoints near sites 1581 and 2152 of the NS5 gene from strains ArD158084, ArB1362 and ArD157995. These findings were confirmed by Bootscan, Maxchi, Chimaera, SiSscan and 3Seq methods and supported by significant *p*-values of 9.93E-9, 3.32E-7, 3.32E-7, 5.27E-28 and 7.65E-24, respectively. These potential recombinant sequences were excluded from further analyses to avoid inferential biases [62], [63]. To perform the phylogenetic analysis we concatenated E and NS5 sequences and replaced inferred recombinant fragments with missing data. This is in line with the use of Maximum Likelihood approaches, which is fairly robust to the introduction of gaps [64], [65]. In addition, we found incompatibilities between E and NS5 phylogenies using GiRaF. The three discordant strains (ArD128000, ArA1465 and ArD142623) were excluded, and we used 40 (31 from E and 36 from NS5) concatenated sequences for phylogenetic analysis. Moreover, we also found that the two remaining datasets for E and NS5 have no conflicting phylogenetic signal, as estimated by a CADM test (*p*-value = 9.99E-5 and α = 0.05). Given the limited sampling that we investigated, these results indicate that ZIKV may be experiencing recombination in the field, which is uncommon among flaviviruses [66]. These findings remain to be properly evaluated and assessed related to their effects on viral spread, zoonotic maintenance and epidemiologic potential. The possibility that our findings could be a consequence of cross contamination among isolates seems highly improbable given the extreme precautions that were taken. RNA extraction and reverse transcription were done separately for each isolate under BSL-II cabinets, sequenced several times leading to identical sequences, even when processed in different laboratories in Sao Paulo, Brazil, and Dakar, Senegal.

### Phylogenetic analysis

**Figure 1:**
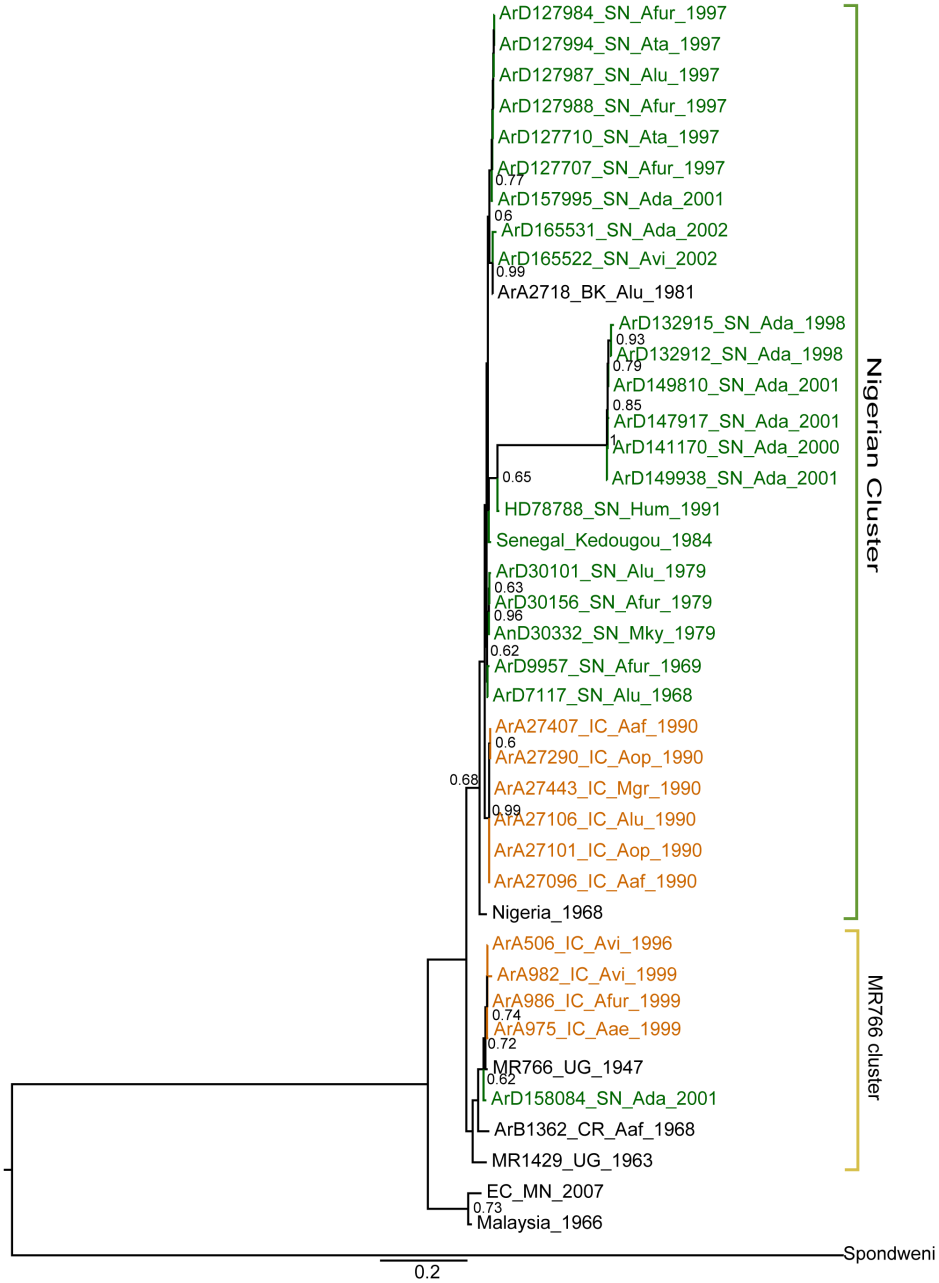
Maximum likelihood phylogenetic tree inferred for concatenated of sequences from Envelope and NS5 genes of Zika virus. Consensus tree summarized after 1000 non-parametric bootstrap replicates, with support values greater than 60% shown in the nodes. The cluster the Ugandan MR766 prototype strain was highlighted by the yellow sector and the Nigerian cluster was highlighted by the green sector. The strains from Senegal and Côte d'Ivoire are shown in green and orange, respectively. The tree has been rooted with the Spondweni lineage isolated in South Africa was used as outgroup to root the tree.

We first investigated the phylogenetic signal content in our data by reconstructing 50000 quartets for each gene segment using the likelihood mapping method (see methods section). Our results indicated that NS5 and E datasets had high phylogenetic signal content given their lower percentage of unresolved quartets (3.2% and 3.4%, respectively), while 3′NCR showed less signal (16.4% of unresolved quartets) and was not considered. The ML trees for E (data not shown), NS5 (data not shown) and the two concatenated genes (Figure 1) reinforced that ZIKV strains could be classified in three major clusters [17]. Accordingly, the African strains were arranged into two groups: the MR766 prototype strain cluster (yellow sector on Figure 1) and the Nigerian cluster (green sector on Figure 1); and the Micronesian and Malaysian strains clustered together forming the Asian clade (Figure 1), in agreement with [26]. For West Africa, the strains from Côte d'Ivoire and Senegal were found in both African clusters, suggesting that at least two distinct lineages of ZIKV circulated in these countries. Interestingly, we found that the position of the Senegalese cluster, comprising viruses isolated from 1998 to 2001 associated with *A. dalzieli*, branching as a sister group of HD78788 isolated in Senegal in 1991, was not simply explained by recombination (with both Giraf and RDP) or poor rooting of the tree, since it did not depend on the inclusion (Figure 1) or exclusion (Figure S2) of the Spondweni, which is a *bonafide* outgroup. It was observed 65% of the time during a highly stringent maximum likelihood (ML) analysis with GARLI, not taking into account dates of isolation, but crucially it had a posterior probability of one during Bayesian Inference (BI) that do take into account dates of isolation. Although we cannot rule out systematic topological errors, BI was certainly better informed than ML, since RNA viruses evolve fast, making their times of isolation important parameters for phylogenetic inference. Moreover, since we did not find compositional or codon usage biases in those sequences and in agreement with the consistent BI results, we could not rule out that the long branch length observed was not due to a detected increase of almost 10 fold increase in the rate of change along that lineage, which was not caused by detectable positive selection, as evaluated using HyPhy.

### Selection analyses

Selection analyses of E and NS5 genes uncovered several sites under strong negative selection indicated by ω<0. This suggests frequent purging of deleterious polymorphisms in functionally important genes. Likewise, the lack of positively selected sites, indicated by ω>0, is typical of highly adapted phenotypes and shows no detectable directional change on the available data. Our findings were expected, as the infection and transmission modes of ZIKV allow the accumulation of synonymous mutations and negatively selected sites [67]. The alternation between arthropod vector and mammal hosts may impose several barriers to non-synonymous mutations in important genes [68].

### Phylodynamic analyses

The μ and the highest posterior densities (HPD with 95% of confidence interval) estimated with Beast for E, NS5 and 3′NCR genomic regions were, respectively, 2.135E-3 (2.04E-3 to 2.33221E-3), 7.1789E-4 (6.9466E-4 to 7.417E-4) and 1.1285E-3 (2.708E-4 to 2.504E-3) substitutions per site per year, which are similar to μ estimated other flaviviruses [69]. As evolutionary rates are the result of spontaneous mutations followed by selection, differences *per gene* are expected and in accordance with their biological role, given that the NS5 is a polymerase and the E is a surface protein. In addition, the root date estimates and 95% HPDs of the phylogenetic trees for E, NS5 and 3′NCR genomic regions were, respectively, 1900 (1851 to 1937), 1927 (1887 to 1940) and 1923 (1874 to 1959). These dates suggest a recent origin for the ZIKV strains (included in this study) near to the beginning of the 20^th^ century.

### Movement of ZIKV

**Figure 2:**
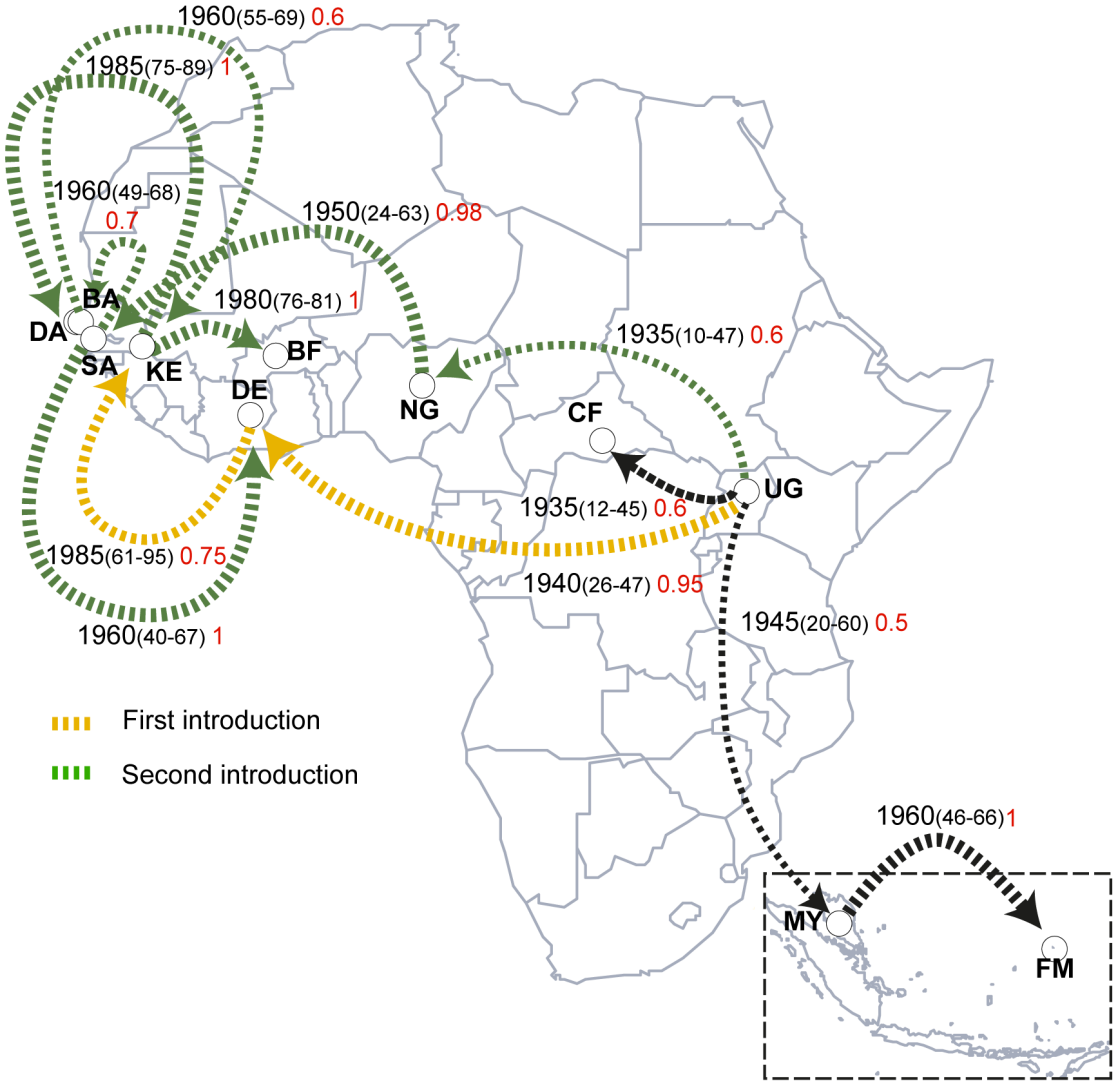
Geographic spread of ZIKV in Africa and Asia. The directed lines connect the most probable sources and target localities of viral lineages (shown by arrows), with widths proportional to the posterior probabilities and values shown in red. Only plausible routes with probabilities above 50% are shown. The distinct introductions into Senegal and Côte d'Ivoire were represented by different colors. The estimated time to the most recent common ancestor of strains from different countries are shown with 95% posterior time intervals in parenthesis and could be interpreted as the oldest possible year of introduction of that lineage at that locality.

Based on our samples we inferred the most likely geographical pathway connecting ZIKV lineages. These results indicated that ZIKV emerged in Uganda around 1920, most probably between 1892 and 1943. This inference is in line with the first known ZIKV isolation in Uganda in 1947 [5]. We found two independent ZIKV introductions into West Africa from the Eastern portion of the continent (Figures 2 and S2A, and kml file in Dataset S1). The first viral introduction into Côte d'Ivoire (CI) and Senegal (SN) was related to the MR766 cluster (yellow lines in Figure 2), which possibly moved from Uganda around 1940 into Dezidougou (CI). From there, this lineage probably spread to Kedougou in Senegal (SN) around 1985 and to Sokala-Sobara (CI) around 1995. The second introduction was related to a Nigerian cluster (green lines in Figure 2), when ZIKV probably moved from Uganda to the Central African Republic and Nigeria around 1935. From Nigeria, the virus probably spread to Saboya (SN) around 1950 and from there to Dezidougou (CI) and Bandia (SN) around 1960. From Bandia, ZIKV was introduced into Kedougou (SN) around 1965 and from there to Burkina Faso around 1980 and to Dakar (SN) around 1985. Moreover, an additional ZIKV lineage from Uganda probably spread to Malaysia around 1945 and from there, the virus reached Micronesia around 1960, forming the Asian cluster [26]. The correlation between viral location (coded as character states) and phylogenies was strongly supported by significant AI and PS values, *p*-values≤1.00 E-4 (Dataset S2). Thus, assuming an origin of ZIKV in Uganda, our findings revealed at least two independent exits from East Africa in agreement with the two previously proposed African clades [17] and also pointed to a viral migratory flow from Eastern Africa to Asia. Although our sampling was the most comprehensive to this date, our conclusions about ZIKV movement are informed conjectures at best on the most plausible hypotheses on ZIKV spreading patterns, which are limited by the inherent biases of this type of analyses.

### Animal sources of ZIKV

The association of the animal sources with viral lineages (Figure S2B) suggested that ZIKV dispersed widely among distinct animal species without a clear pattern of preference, maybe associated to the enzootic cycle of ZIKV in Africa, whose natural cycle allows a broad range of hosts [70]. Nevertheless, we found significant MC (*p*-value≈1.00 E-4, Dataset S2) for ZIKV strains isolated from *A. dalzieli*, suggesting a possible important role of this zoophilic vector [71] in West Africa. This association was found to be robust to the exclusion of vertebrate host from the analysis. The plausibility of the putative recombination events we detected (Table S2), could in part be explained by mosquitoes taking sequential blood meals from distinct animal species harboring distinct ZIKV lineages, which is in line with ours and others host range findings [70]. Also, when analyzing the increase of ZIKV activity in Kedougou, (where most of the viruses analyzed herein were collected), we noticed that ZIKV activity is much more frequent, with an interval of 1–2 years, compared to the 5 to 8 years cycle of dengue and yellow fever virus. Hence from 1972 to 2002, ZIKV emerged in 1973, 1976, 1979, 1980 and 1981. Such frequent activity can also be an opportunity of co-circulation and mixing of multiple genotypes present in the forest and that may favor recombination among them.

### A phylodynamic context for recombination events

The occurrence of recombination among ZIKV strains in time-scaled phylogenetic trees suggested that some ZIKV lineages sampled in Dezidougou (CI) in 1990 (ArA27101, ArA27290, ArA27096, ArA27443, ArA27407 and ArA27106) with recombinant E (Figure S2C) shared a common ancestor around 1962 (ranging from 1951 to 1967 HPD with 95% of confidence interval). Likewise, the strain ArA982 was also isolated at Dezidougou in 1999 and its sister-group ArA986, which shared a common ancestor with the former around 1992 (ranging from 1981 to 1996 HPD with 95% of confidence interval), was sampled in the neighbor province Sokala-Sobara (CI) in 1999. Together these results indicated that recombination in envelope protein could be an important trend among the enzootic cycle of ZIKV at this region in Côte d'Ivoire, as ZIKV lineages did not show a clear pattern of host preference and recombination requires the infection of the same host by more than one viral strain. Besides, the other E recombinant strain (HD78788), isolated from a human case at Dakar (SN) in 1991, shared a common ancestor around 1984 (ranging from 1976 to 1988 HPD with 95% of confidence interval) with ZIKV strains from Kedougou (SN). Conversely, the three NS5 recombinants did not cluster along phylogenetic trees (Figure S2C), although two of them were isolated in Kedougou from *A. dalzieli* mosquitoes in 2001 (ArD157995 and ArD158084) and the other (ArB1362) was isolated in Bouboui, Central African Republic, from *A. africanus* mosquitoes in 1968. The preferential distribution of recombinant strains along phylogenies was supported by significant *p*-values of AI and PS ≤2.00E-4 (Dataset S2) and the adjacency patterns of E and NS5 recombinants were also confirmed by MC statistics (Dataset S2).

### Glycosylation patterns in ZIKV envelope protein

**Figure 3:**
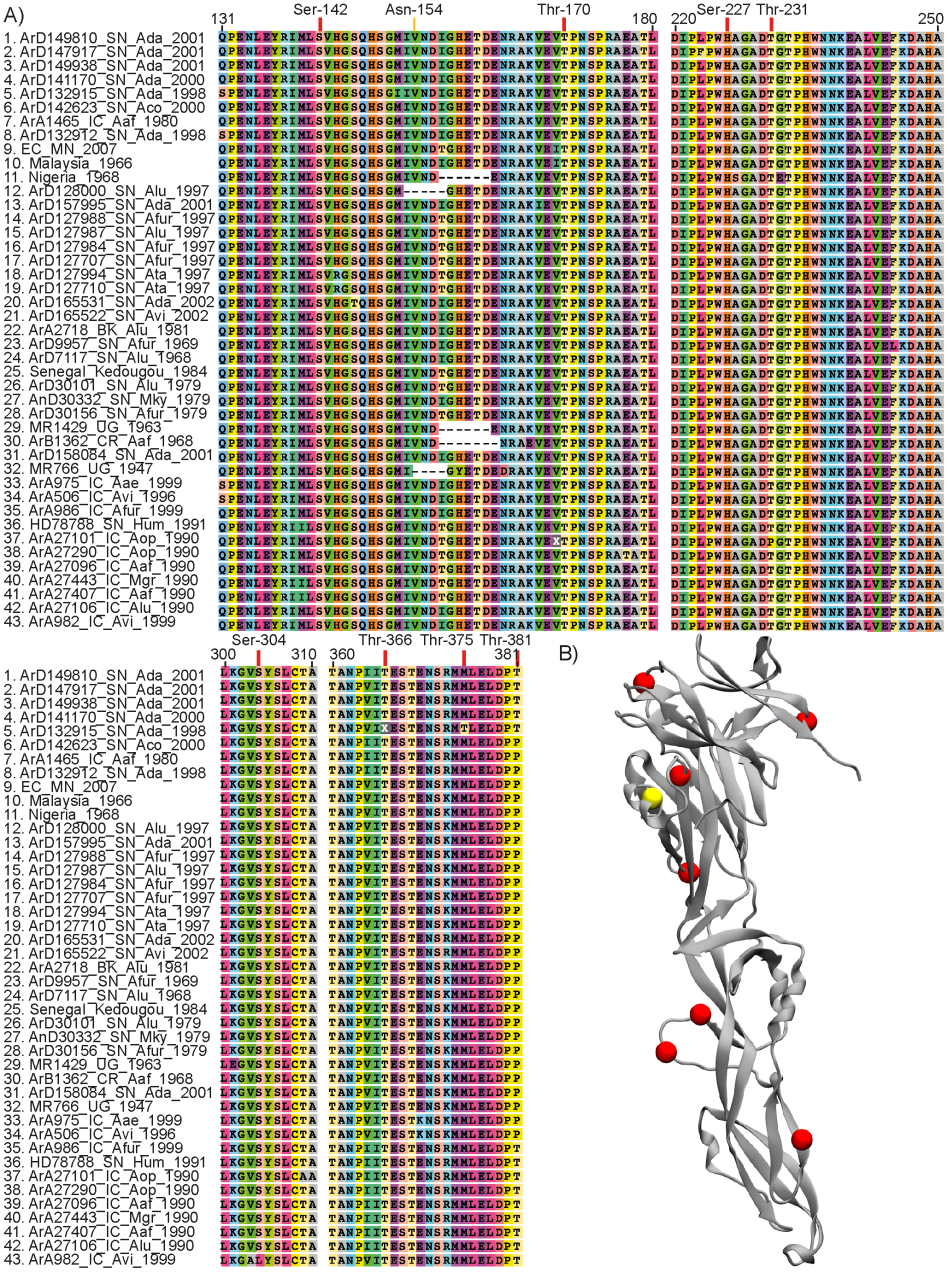
Mapping of predicted glycosylation sites on envelope protein of ZIKV. A) Alignment of E protein showing predicted glycosylation sites. Red arrows point to O-linked glycosylation sites (Ser or Thr residues) and the yellow arrow points to the N-linked glycosylation site (Asn-X-Thr motif). B) Tridimensional structure of E protein. Red beads indicate O-linked glycosylation sites and the yellow bead indicates the unique N-linked glycosylation site.

Our analyses predicted several glycosylation sites in the E protein (Figure 3). We detected a probable motif (Asn-X-Thr) among E sequences from several ZIKV strains (Figure 3A), which suggests a N-linked glycosylation site in the residue Asn-154, in agreement with [17], [26]. This residue is located on an α-helix in the E protein structure (yellow arrow in Figure 3A and yellow bead in Figure 3B). Our results also pointed several O-linked glycosylation sites in the E protein (red arrows in Figure 3A and red beads Figure 3B) but no C-mannosylated site. We found a probable mucin-type O-linked glycosylated site at residue Thr-170 in E protein from all ZIKV strains, and other mucin sites at residues Thr-245 and Thr-381 in some isolates (Figure 3A). In addition, we also uncovered probable O-GlcNAc attachment sites at residues Ser-142, Ser-227, Thr-231, Ser-304, Thr-366 and Thr-381 in E from some strains (Figure 3A).

Given the importance of the N-linked glycosylation site around position 154 of the E protein for infectivity and assembly of flaviviruses [72]–[74] and the fact that we observed polymorphisms in this motif (deletions and substitutions 156 Thr/Iso), we investigated the correlation between the conservation of this motif (Asn-X-Thr) and phylogenies for ZIKV strains. Our results suggested that the acquisition of this glycosylation site is a recurrent event in the history of ZIKV, given the observed changes from Isoleucine to Threonine and vice-versa more than once in the MCC tree (Figure S2D), supported by *p*-values for AI and PS ≤7.00E-4 (Dataset S2). However, our conclusions are limited due to serial passages of the former ZIKV strains (Figure S2D) in mouse brain [26], which could result in the loss of this glycosylation site, as observed in West Nile virus [75].

### Correlated evolutionary change along ZIKV phylogenies

Since it was demonstrated that the absence of an N-linked glycosylation site on the E protein enhances viral infectivity for C6/36 mosquito cells [72], [73] and E protein of ZIKV strains from *A. dalzieli*, which was the unique vector source with significant MC–showed an absence of this glycosylation site, we investigated the correlation between this mosquito-source and N-linked glycosylation patterns of E protein along PST. Our results indicated the changes in glycosylation patterns (presence or absence) and vector (*A. dalzieli* or not) were correlated during ZIKV emergence, which was supported by BF for dependent model (BF≈47.004) greater than for them to independent model. These findings could be related to the enzootic cycle of ZIKV in West Africa and the zoophilic behavior of *A. dalzieli* [71], whose females take blood meals from a broad range of vertebrates, which provides additional evidence for the absence of host preference (as described in Animal sources of ZIKV). Hence, further studies are necessary to understand the consequences of our results to ZIKV transmission cycle in nature.

### Biological correlates of our findings

Our analyses indicated that ZIKV may have experienced several recombination events, which is uncommon among flaviviruses [66]. The recurrent loss and gain of the N-linked glycosylation site in the E protein could be related to mosquito-cell infectivity [73] and the correlated loss of this glycosylation site in ZIKV strains from *A. dalzieli* also provides indirect evidence for the enzootic cycle, since this vector has a zoophilic behavior [71] that may spread ZIKV among several hosts. Crucially, our results corroborated the notion that at least three distinct ZIKV clusters shared a common ancestor possibly with Ugandan lineages around 1920, followed by two events of East to West Africa spread (Figure 2): (*i*) one related to the MR766 cluster introduction to Côte d'Ivoire and posterior spread to Senegal and; (*ii*) other related to the Nigerian cluster introduction in Senegal and posterior dispersion to Côte d'Ivoire and Burkina Faso.

## Supporting Information

Dataset S1 **Spread of ZIKV strains in Africa and Asia.** A kml file to picture the history of ZIKV movement into Africa and Asia during the time, it is executable in Google Earth program (http://www.google.com/earth/).
(KML)

Dataset S2 **Significance of the correlation among phylogenies and attributes of ZIKV lineages.**
(DOC)

Figure S1 **Recombination analysis using Rec-HMM along ZIKV genomes.** The dashed green lines indicate estimated breakpoints in the genomes.
(TIF)

Figure S2 **Maximum clade credibility (MCC) trees for concatenated sequences summarizing lineage states along a time-scaled tree, with posterior probability values shown near the nodes.** (**A**) Most probable geographical location coded according to map (Figure 2): Uganda (UG), Central African Republic (CF), Dezidougou in Côte d'Ivoire (DE), Sokala-Sobara in Côte d'Ivoire (SS), Kedougou in Senegal (KE), Saboya in Senegal (SA), Bandia in Senegal (BA), Dakar in Senegal (DA), Burkina Faso (BF), Nigeria (NG), Malaysia (MY) and Yap Island in the Federated States of Micronesia (FM); (**B**) most probable animal source; (**C**) recombination events per region; and (**D**) glycosylation polymorphisms.
(TIF)

Table S1 **Source, country and year of isolation from ZIKV strains used in this study.**
(DOC)

Table S2 **Detection of recombination events in ZIKV genomes.**
(DOC)
