# Supplementary material for: Molecular Evolution of Zika Virus during Its Emergence in the 20th Century
Source: PLoS Negl Trop Dis. 2014 Jan 9;8(1):e2636. doi: 10.1371/journal.pntd.0002636 (PMC3888466; doi:10.1371/journal.pntd.0002636)
Supplement: Dataset S2 — Significance of the correlation among phylogenies and attributes of ZIKV lineages. (DOC) [file pntd.0002636.s002.doc]

**Significance of the correlation among ZIKV phylogenies and locality from viral sampling**

| **Statistic** | **Observed mean** | **Confidence interval (95%)** | **Null mean** | **Confidence interval (95%)** | ***p*-value** |
| --- | --- | --- | --- | --- | --- |
| AI | 0.93 | 0.61-1.25 | 2.98 | 2.45-3.48 | 0 |
| PS | 13.00 | 13.0-13.0 | 18.87 | 17.23-19.96 | 0 |
| MC  Kedougou | 7.0 | 7.0-7.0 | 2.86 | 2.01-4.58 | 0 |
| MC  Dezidougou | 6.0 | 6.0-6.0 | 1.50 | 1.0-2.26 | 1.00E-4 |
| MC  Burkina Faso | 1.0 | 1.0-1.0 | 1.0 | 1.0-1.0 | 1.0 |
| MC  Sokala-Sobara | 1.0 | 1.0-1.0 | 1.0 | 1.0-1.0 | 1.0 |
| MC  Central African Republic | 1.0 | 1.0-1.0 | 1.0 | 1.0-1.0 | 1.0 |
| MC  Saboya | 1.0 | 1.0-1.0 | 1.0 | 1.0-1.0 | 1.0 |
| MC  Bandia | 1.0 | 1.0-1.0 | 1.0 | 1.0-1.0 | 1.0 |
| MC  Micronesia | 1.0 | 1.0-1.0 | 1.0 | 1.0-1.0 | 1.0 |
| MC  Dakar | 1.0 | 1.0-1.0 | 1.0 | 1.0-1.0 | 1.0 |
| MC  Uganda | 1.0 | 1.0-1.0 | 1.01 | 1.0-1.0.01 | 1.0 |
| MC  Malaysia | 1.0 | 1.0-1.0 | 1.0 | 1.0-1.0 | 1.0 |
| MC  Nigeria | 1.0 | 1.0-1.0 | 1.0 | 1.0-1.0 | 1.0 |

**Significance of the correlation among ZIKV phylogenies and animal-source from viral sampling**

| **Statistic** | **Observed mean** | **Confidence interval (95%)** | **Null mean** | **Confidence interval (95%)** | ***p*-value** |
| --- | --- | --- | --- | --- | --- |
| AI | 3.45 | 3.04-3.84 | 3.90 | 3.44-4.24 | 0.06 |
| PS | 26.05 | 25-27 | 28.36 | 26.27-30.11 | 0.03 |
| MC  Monkey | 1.00 | 1.0-1.0 | 1.04 | 1-1.2 | 1.0 |
| MC  A. africanus | 1.00 | 1.0-1.0 | 1.09 | 1.0-1.71 | 1.0 |
| MC  A. opock | 1.00 | 1.0-1.0 | 1.02 | 1.0-1.05 | 1.0 |
| MC  A. luteocephalus | 1.0 | 1.0-1.0 | 1.14 | 1.0-1.92 | 1.0 |
| MC  A. grahami | 1.0 | 1.0-1.0 | 1.0 | 1.0-1.0 | 1.0 |
| MC  A. vittatus | 1.05 | 1.0-1.0 | 1.05 | 1.0-1.24 | 1.0 |
| MC  A. aegypti | 1.0 | 1.0-1.0 | 1.01 | 1.0-1.04 | 1.0 |
| MC  A. furcifer | 1.01 | 1.0-1.0 | 1.22 | 1.0-2.00 | 1.0 |
| MC  A. taylori | 1.00 | 1.0-1.0 | 1.01 | 1.0-1.01 | 1.0 |
| MC  A. dalzieli | 6.0 | 6.0-6.0 | 1.50 | 1.0-2.28 | 1.00E-4 |
| MC  Human | 1.0 | 1.0-1.0 | 1.04 | 1.0-1.24 | 1.0 |

**Significance of the correlation among ZIKV phylogenies and distribution of recombinant strains**

| **Statistic** | **Observed mean** | **Confidence interval (95%)** | **Null mean** | **Confidence interval (95%)** | ***p*-value** |
| --- | --- | --- | --- | --- | --- |
| AI | 0.65 | 0.23-0.99 | 1.91 | 1.36-2.42 | 2.00E-4 |
| PS | 6.78 | 6.0-7.0 | 11.19 | 9.85-11.99 | 2.00E-4 |
| MC  Non-recombinant | 6.00 | 6.0-6.0 | 4.63 | 2.93-7.86 | 0.30 |
| MC  E-recombinant | 6.00 | 6.0-6.0 | 1.51 | 1.00-2.29 | 2.00E-4 |
| MC  NS5-recombinant | 1.00 | 1.00-1.00 | 1.05 | 1.0-1.25 | 1.00 |

**Significance of the correlation among ZIKV phylogenies and pattern of N-linked glycosylation site in envelope**

| **Statistic** | **Observed mean** | **Confidence interval (95%)** | **Null mean** | **Confidence interval (95%)** | ***p*-value** |
| --- | --- | --- | --- | --- | --- |
| AI | 0.89 | 0.49-1.28 | 2.45 | 1.81-3.06 | 0 |
| PS | 10.56 | 10.0-11.0 | 15.89 | 13.49-18.02 | 7.00E-4 |
| MC Isoleucine | 6.0 | 6.0-6.0 | 2.85 | 2.00-4.62 | 0.03 |
| MC Threonine | 3.89 | 2.0-6.0 | 2.33 | 1.49-3.36 | 0.02 |
| MC  Deletion | 1.00 | 1.00-1.00 | 1.09 | 1.0-1.66 | 1.0 |
