# Supplementary figures and images for: Molecular Evolution of Zika Virus during Its Emergence in the 20th Century
Source: PLoS Negl Trop Dis. 2014 Jan 9;8(1):e2636. doi: 10.1371/journal.pntd.0002636 (PMC3888466; doi:10.1371/journal.pntd.0002636)

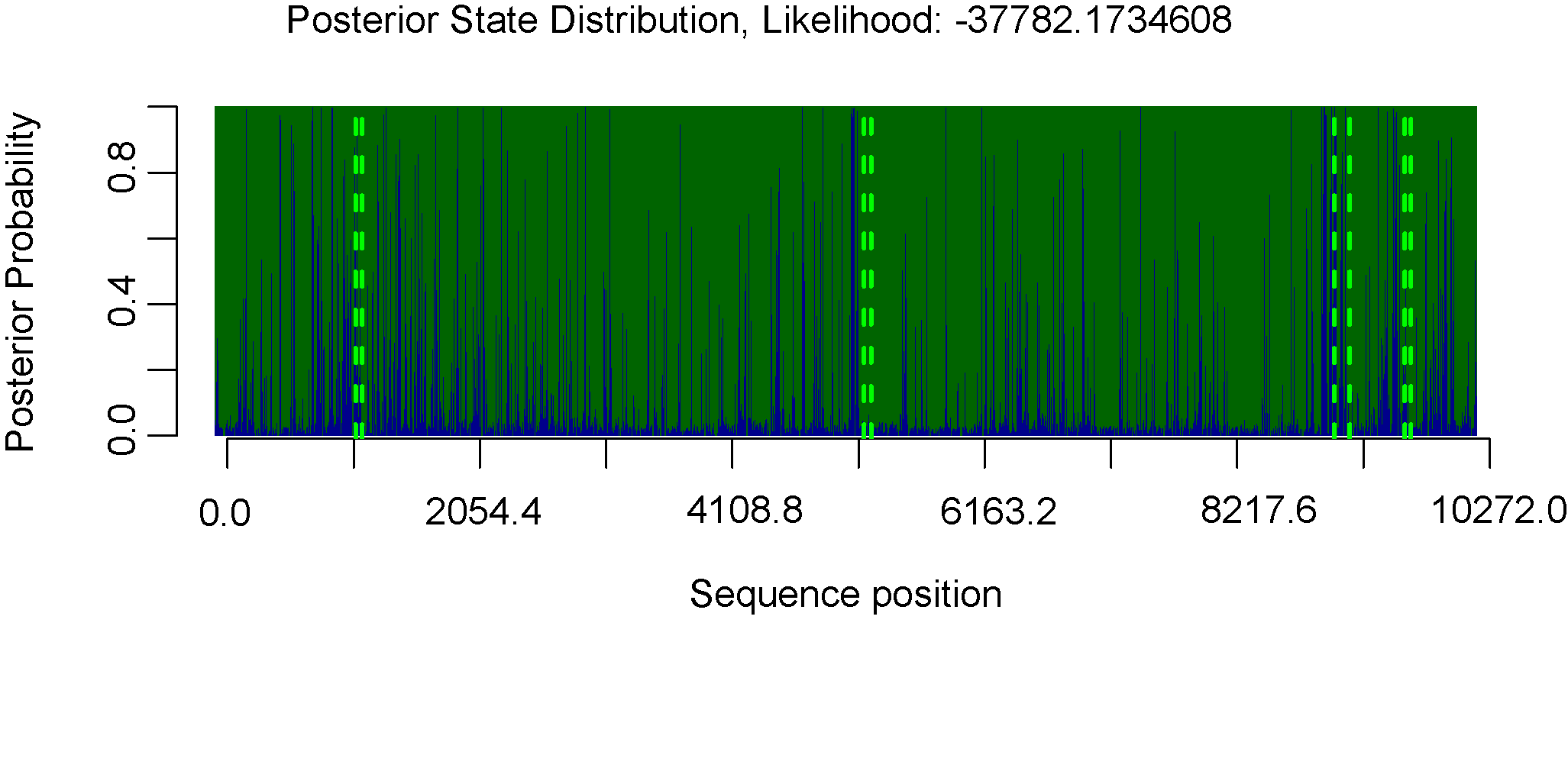

Supplement: Figure S1 — Recombination analysis using Rec-HMM along ZIKV genomes. The dashed green lines indicate estimated breakpoints in the genomes. (TIF) [file pntd.0002636.s003.tif]

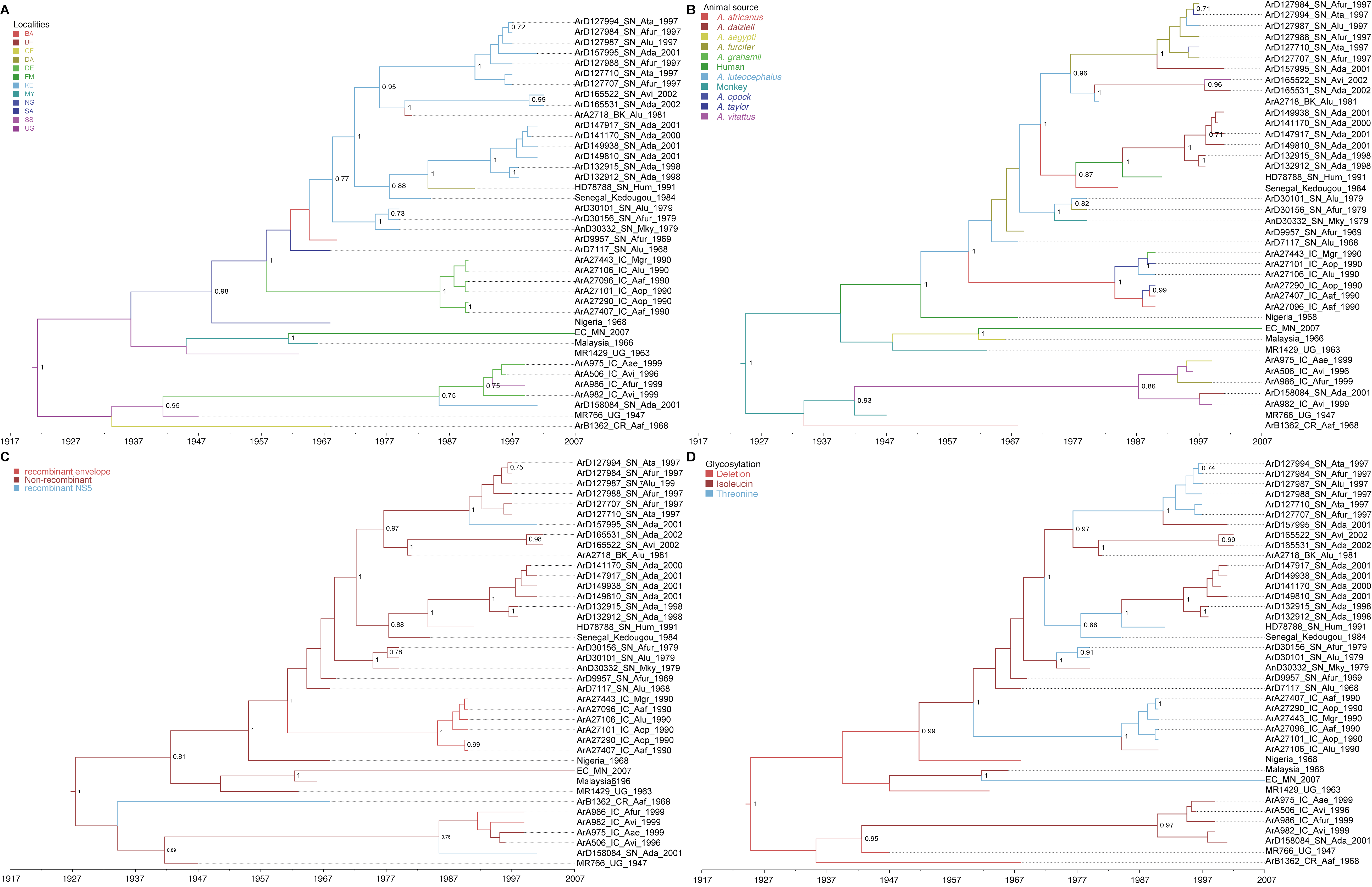

Supplement: Figure S2 — Maximum clade credibility (MCC) trees for concatenated sequences summarizing lineage states along a time-scaled tree, with posterior probability values shown near the nodes. (A) Most probable geographical location coded according to map (Figure 2): Uganda (UG), Central African Republic (CF), Dezidougou in Côte d'Ivoire (DE), Sokala-Sobara in Côte d'Ivoire (SS), Kedougou in Senegal (KE), Saboya in Senegal (SA), Bandia in Senegal (BA), Dakar in Senegal (DA), Burkina Faso (BF), Nigeria (NG), Malaysia (MY) and Yap Island in the Federated States of Micronesia (FM); (B) most probable animal source; (C) recombination events per region; and (D) glycosylation polymorphisms. (TIF) [file pntd.0002636.s004.tif]
