# Supplementary material for: Molecular Evolution of Zika Virus during Its Emergence in the 20th Century
Source: PLoS Negl Trop Dis. 2014 Jan 9;8(1):e2636. doi: 10.1371/journal.pntd.0002636 (PMC3888466; doi:10.1371/journal.pntd.0002636)
Supplement: Table S1 — Source, country and year of isolation from ZIKV strains used in this study. (DOC) [file pntd.0002636.s005.doc]

| Strain | Source | Countries | Year |
| --- | --- | --- | --- |
| ArD 7117 | *Aedes luteocephalus* | Senegal | 1968 |
| ArD 9957 | *Aedes furcifer* | Senegal | 1969 |
| ArD30101 | *Aedes luteocephalus* | Senegal | 1979 |
| ArD 30156 | *Aedes furcifer* | Senegal | 1979 |
| AnD 30332 | *Cercopithecus aethiops* | Senegal | 1979 |
| HD 78788 | *Homo sapiens* | Senegal | 1991 |
| ArD 127707 | *Aedes furcifer* | Senegal | 1997 |
| ArD 127710 | *Aedes taylori* | Senegal | 1997 |
| ArD 127984 | *Aedes furcifer* | Senegal | 1997 |
| ArD 127987 | *Aedes luteocephalus* | Senegal | 1997 |
| ArD 127988 | *Aedes furcifer* | Senegal | 1997 |
| ArD 127994 | *Aedes taylori* | Senegal | 1997 |
| ArD 128000 | *Aedes luteocephalus* | Senegal | 1997 |
| ArD 132912 | *Aedes dalzieli* | Senegal | 1998 |
| ArD 132915 | *Aedes dalzieli* | Senegal | 1998 |
| ArD 141170 | *Aedes dalzieli* | Senegal | 2000 |
| ArD 142623 | *Anopheles coustani* | Senegal | 2000 |
| ArD 149917 | *Aedes dalzieli* | Senegal | 2001 |
| ArD 149810 | *Aedes dalzieli* | Senegal | 2001 |
| ArD 149938 | *Aedes dalzieli* | Senegal | 2001 |
| ArD 157995 | *Aedes dalzieli* | Senegal | 2001 |
| ArD 158084 | *Aedes dalzieli* | Senegal | 2001 |
| ArD 165522 | *Aedes vittatus* | Senegal | 2002 |
| ArD 165531 | *Aedes dalzieli* | Senegal | 2002 |
| ArA 1465 | *Aedes africanus* | Côte d’Ivoire | 1980 |
| ArA 27101 | *Aedes opok* | Côte d’Ivoire | 1990 |
| ArA 27290 | *Aedes opok* | Côte d’Ivoire | 1990 |
| ArA 27106 | *Aedes luteocephalus* | Côte d’Ivoire | 1990 |
| ArA 27096 | *Aedes africanus* | Côte d’Ivoire | 1990 |
| ArA 27407 | *Aedes africanus* | Côte d’Ivoire | 1990 |
| ArA 27443 | *Aedes (Mucidus) grahamii* | Côte d’Ivoire | 1990 |
| ArA 506/96 | *Aedes vittatus* | Côte d’Ivoire | 1996 |
| ArA 975-99 | *Aedes aegypti* | Côte d’Ivoire | 1999 |
| ArA 982-99 | *Aedes vittatus* | Côte d’Ivoire | 1999 |
| ArA 986-99 | *Aedes furcifer* | Côte d’Ivoire | 1999 |
| ArA 2718 | *Aedes luteocephalus* | Burkina Faso | 1981 |
| ArB 1362 | *Aedes africanus* | Central African Republic | 1968 |
| Nigeria68 | *Homo sapiens* | Nigeria | 1968 |
| Malaysia66 | *Aedes aegypti* | Malaysia | 1966 |
| Kedougou84 | *Aedes africanus* | Senegal | 1984 |
| MR766 | *Macaca mulatta* | Uganda | 1947 |
| MR1429 | *Macaca mulatta* | Uganda | 1963 |
| ECMN2007 | *Homo sapiens* | Micronesia | 2007 |
