# Supplementary material for: Molecular Evolution of Zika Virus during Its Emergence in the 20th Century
Source: PLoS Negl Trop Dis. 2014 Jan 9;8(1):e2636. doi: 10.1371/journal.pntd.0002636 (PMC3888466; doi:10.1371/journal.pntd.0002636)
Supplement: Table S2 — Detection of recombination events in ZIKV genomes. (DOC) [file pntd.0002636.s006.doc]

| **Begin** | **End** | **Recombinants** | **RDP** | **GENECONV** | **Bootscan** | **Maxchi** | **Chimaera** | **SiSscan** | **3Seq** |
| --- | --- | --- | --- | --- | --- | --- | --- | --- | --- |
| 3119 | 4261 | ArD142623 | 5.66E-38 | 1.34E-37 | 6.35E-42 | 2.79E-17 | 1.69E-16 | 5.54E-14 | NS^#^ |
| 93* | 805 | ArD128000 | 1.76E-33 | 5.25E-34 | 1.42E-33 | 5.69E-05 | 3.79E-13 | 1.37E-14 | 4.83E-11 |
| 1352 | 1835 | ArD157995 | 2.10E-33 | 1.07E-28 | 3.98E-18 | 1.10E-09 | 9.02E-10 | 2.43E-10 | 3.84E-09 |
| 5161 | 5222 | ArD157995 | 2.39E-28 | 1.19E-22 | NS^#^ | 1.33E-09 | 2.39E-09 | NS^#^ | NS^#^ |
| 8271 | 9002 | ArD142623 | 3.05E-18 | 5.69E-28 | 3.25E-21 | 2.91E-13 | 4.27E-11 | 2.22E-21 | NS^#^ |
| 5096 | 5555* | ArD128000 | 3.83E-25 | 3.76E-23 | 3.13E-25 | 6.35E-10 | 2.82E-10 | 4.62E-11 | 2.01E-03 |
| 5096 | 5555* | ArD7117 | 3.83E-25 | 3.76E-23 | 3.13E-25 | 6.35E-10 | 2.82E-10 | 4.62E-11 | 2.01E-03 |
| 3003 | 3530 | ArD128000 | 1.12E-23 | 1.32E-21 | 9.45E-24 | 1.91E-08 | 9.25E-09 | 4.18E-09 | 2.89E-05 |
| 847 | 1272 | ArD158084 | 1.34E-15 | 5.94E-13 | 3.99E-16 | 1.85E-08 | 8.28E-07 | 7.55E-04 | NS^#^ |
| 847 | 1272 | ArD157995 | 1.34E-15 | 5.94E-13 | 3.99E-16 | 1.85E-08 | 8.28E-07 | 7.55E-04 | NS^#^ |
| 5631* | 6498 | ArD142623 | 5.58E-23 | 2.31E-22 | 1.12E-24 | 1.21E-13 | 3.17E-12 | 1.99E-19 | NS^#^ |
| 1* | 634 | ArD142623 | 5.71E-24 | 1.35E-21 | 1.33E-14 | 7.65E-05 | 9.08E-05 | 2.04E-15 | NS^#^ |
| 9560 | 9802 | ArD142623 | 1.77E-12 | 1.25E-11 | 4.63E-13 | 8.56E-05 | 2.75E-06 | NS^#^ | NS^#^ |
| 9003* | 9176 | ArD157995 | 1.77E-11 | 2.15E-06 | 2.78E-06 | 6.08E-04 | NS^#^ | NS^#^ | NS^#^ |
| 9003* | 9176 | ArD158084 | 1.77E-11 | 2.15E-06 | 2.78E-06 | 6.08E-04 | NS^#^ | NS^#^ | NS^#^ |
| 7172 | 7406 | ArD142623 | 2.87E-06 | 1.68E-09 | 9.18E-08 | 1.12E-06 | 3.02E-06 | 9.75E-07 | NS^#^ |

*The actual breakpoint is undetermined. It was most likely overprinted by a subsequent recombination event.

^#^No significant *p*-value was recorded for this recombination event using this method.
